# Evaluation of selected semen parameters and biomarkers of male infertility – preliminary study

**DOI:** 10.12688/f1000research.121622.1

**Published:** 2022-05-30

**Authors:** Michal Kups, Kamil Gill, Aleksandra Rosiak-Gill, Patryk Harasny, Tomasz Machalowski, Marta Grabowska, Rafal Kurzawa, Olimpia Sipak, Malgorzata Piasecka

**Affiliations:** 1 The Fertility Partnership Vitrolive in Szczecin, Szczecin, West Pomeranian Voivodeship, 70-483, Poland; 2 Department of Urology and Oncological Urology, Regional Specialist Hospital in Szczecin, Szczecin, West Pomeranian Voivodeship, 71-455, Poland; 3 Department of Histology and Developmental Biology, Faculty of Health Sciences, Pomeranian Medical University in Szczecin, Szczecin, West Pomeranian Voivodeship, 71-210, Poland; 4 Department of Urology and Urological Oncology, Faculty of Medicine and Dentistry, Pomeranian Medical University in Szczecin, Szczecin, West Pomeranian Voivodeship, 70-111, Poland; 5 Department of Perinatology, Obstetrics and Gynecology, Faculty of Medicine and Dentistry, Pomeranian Medical University in Szczecin, Police, West Pomeranian Voivodeship, 72-010, Poland; 6 Department of Gynecology and Reproductive Health, Faculty of Health Sciences, Pomeranian Medical University in Szczecin, Szczecin, West Pomeranian Voivodeship, 71-210, Poland; 7 Department of Obstetrics and Pathology of Pregnancy, Faculty of Health Sciences, Pomeranian Medical University in Szczecin, Szczecin, Szczecin, West Pomeranian Voivodeship, 71-210, Poland

**Keywords:** male infertility, semen characteristics, testicular volume, reproductive hormones, sperm nuclear DNA integrity

## Abstract

**Background:** Because the etiopathogenesis of male infertility is multifactorial our study was designed to clarify the relationship between standard semen parameters, testicular volume, levels of reproductive hormones and the fragmentation of sperm nuclear DNA (SDF).

**Methods:** Patients (n = 130) were clustered as subjects: 1) with an abnormal volume (utrasonography) of at least one testis (<12 mL) or with a normal volume of testes and 2) with abnormal levels of at least one of the reproductive hormones (FSH, LH, PRL, TSH, total T – electrochemiluminescence method) or with normal hormonal profiles and 3) with high level of SDF (>30%), moderate (>15–30%) or low (≤15%) (sperm chromatin dispersion test).

**Results:** In subjects with a decreased testicular volume and in subjects with abnormal levels of reproductive hormones, decreased basic semen parameters were found. Participants with abnormal testicular volume had a higher percentage of SDF and a higher level of FSH (Mann–Whitney U test). In turn, men with a high level of SDF had lower testicular volume and conventional sperm parameters than men with a low level of SDF (Kruskal–Wallis test).

**Conclusions**: We showed that spermatogenesis disorders coexisted with decreased testicular volume and increased FSH levels. The disorders of spermatogenesis were manifested by reduced basic sperm characteristics and a high level of sperm nuclear DNA damage.

## Introduction

The etiopathogenesis of male infertility is a multifactorial medical problem and is correlated with many congenital and acquired defects of the urogenital tract, cancers, urogenital infections, heat stress in the scrotum, hormonal disorders, genetic abnormalities and immunological factors. It is estimated that approximately 30–50% of male infertility cases are recognized as idiopathic, very often associated with low-quality of spermatozoa. ^1^^–^^4^ On the other hand, unexplained infertility (couples where male patients have normal basic semen parameters and female patients have normal ovulation and fallopian tube potency) is diagnosed in 15–30% of cases. ^1^^–^^4^ Therefore, the comprehensive evaluation of male fertility status should be developed using scrotal ultrasonography (USG) and assessment of the key reproductive hormone as well as advanced seminological tests. ^3^^,^^5^^–^^12^

Available data has suggested that it is possible that infertile men could have normal standard semen characteristics. ^1^^,^^13^^,^^14^ Therefore, it is important to look beyond conventional semen analysis. Many authors report that among the advanced sperm tests, the assays that verify sperm nuclear DNA fragmentation (SDF) are the most clinically useful. Furthermore, evaluation of the percentage of SDF could significantly help in determining the most beneficial treatment algorithm for couples trying to have offspring. ^4^^,^^5^^,^^7^^,^^15^^–^^20^ An SDF ≤15% is considered a normal value (low level of nuclear DNA damage) and correlates with high male fertility potential. In these cases, the chance of becoming pregnant naturally or by intrauterine insemination (IUI) is high. In turn, SDF >15–30% (moderate level of DNA damage) can be associated with a reduced chance of becoming pregnant through natural conception and IUI or even *in vitro* fertilization (IVF) treatment. This range of SDF values and history of previous unsuccessful attempts to achieve pregnancy might indicate the need to introduce intracytoplasmic sperm injection (ICSI). Finally, a high level of SDF (>30%) is strongly associated with a significantly increased risk of reproductive failure, including ICSI treatment. It should be highlighted that even if pregnancy due to assisted reproductive technology (ART) is achievable, the percentage of sperm cells with a fragmented genome >30%, especially >40%, may significantly increase the risk of pregnancy loss. ^4^^,^^16^^,^^19^^,^^21^^–^^30^ These three ranges of sperm DNA damage (≤15%, >15–30% and >30) were primarily recommended for interpretation of the SCSA test results, ^16^^,^^22^^,^^25^^,^^26^^,^^29^ however similar ranges also have been successfully adapted to sperm chromatin dispersion test (SCD). ^19^^,^^23^^,^^31^ Hence, an in-depth assessment of male fertility status, including testicular ultrasound, the levels of reproductive hormones and basic and advanced semen analysis, is clinically justified. Therefore, our study was designed to 1) determine the relationship between testicular volume, levels of reproductive hormones (follicle-stimulating hormone – FSH, luteinizing hormone – LH, prolactin – PRL, total testosterone – total T, thyroid-stimulating hormone – TSH), standard semen analysis and sperm genomic integrity and 2) compare standard semen parameters and investigated biomarkers of male infertility between groups of participants with low, moderate and high levels of nuclear DNA fragmentation.

## Methods

### Ethical considerations

In accordance with the Declaration of Helsinki, all participants in the study indicated their written conscious and voluntary consent to participate in the scientific project. The study was conducted according to the guidelines of the Declaration of Helsinki and approved by the Ethics Committee of Pomeranian Medical University, Szczecin, Poland (KB-0012/21/18, date of approval: 5 February 2018).

### Study population

**Figure 1.**
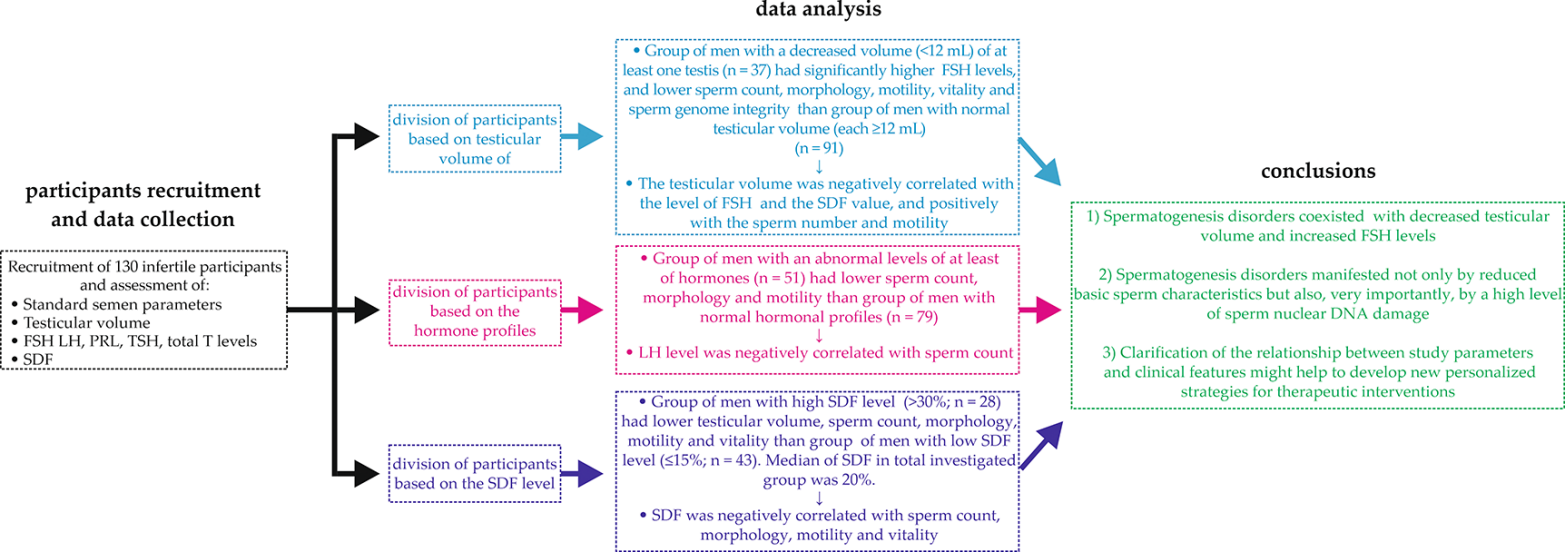
The strategy developed in study analyses. FSH – follicle-stimulating hormone, LH – luteinizing hormone, SDF – sperm DNA fragmentation, PRL – prolactin, TSH – thyroid-stimulating hormone, total T – total testosterone.

The study population consisted of 130 male infertile participants (median age: 33.00 years; range: 23–51 years) who were treated in 2018-2021 in the Individual Specialist Medical Practice (Szczecin, Poland) and The Fertility Partnership Vitrolive in Szczecin (Poland) – Gynaecology and Fertility Clinic and who gave their consent to participate in the study. All patients were partners of women (n = 130; median age: 30.00 years; range: 22–46 years) who did not become pregnant during one year (median: 2 years; range: 1.00–14.00 years) of regular intercourse without contraception ( Figure 1). All initially qualified participants during a medical interview reported to the Laboratory of Andrology in the Department of Histology and Developmental Biology (Pomeranian Medical University, Szczecin, Poland) for seminological analysis. Based on the performed basic semen analysis, men with azoospermia and cryptozoospermia were excluded from the study group.

The infertile status of subjects was verified based on an in-depth medical interview conducted by a specialist in urology (M. K.). The interview included information about factors that may affect fertility potential (genital injuries, cryptorchidism, varicocele, urogenital infections, chronic diseases, pharmacotherapy, use of anabolic steroids, operations and treatments, exposure to harmful factors, lifestyle, stimulants and others) (the interview form can be found as *Extended data.* ^111^ Moreover, the physical medical examination included body assessment (body and hair proportions), and palpation (penis, gonads, epididymides, seminal cords, inguinal canal, prostate and mammary glands) was carried out.

### Conventional semen analysis

Standard semen analysis was carried out according to World Health Organization (WHO) recommendations. ^32^ Semen samples were collected in a sterile urine container by masturbation after a two- to seven-day sexual abstinence. After complete liquefaction of semen (at 37 °C), standard semen analysis was carried out at room temperature – 22°C. The macroscopic evaluation of the semen included color, viscosity, volume and pH. In turn, the microscopic assessment (light/phase-contrast microscope DM500, Lecia, Heerbrugg, Switzerland) included the initial verification of the samples (presence of mucus bands, erythrocytes, epithelial cells, spermine crystals, residual bodies, aggregation and agglutination of sperm) as well as the assessment of the sperm concentration and the total sperm count, motility (progressive and nonprogressive motility), morphology, vitality and the concentration of inflammatory cells. Sperm concentration (analyzed in an improved Neubauer hemocytometer – Heinz Hernez Medizinalbedarf GmbH, Hamburg, Germany, ref no 1080339), sperm motility and vitality (eosin-positive cells and hypoosmotic-reactive cells: HOS test-positive cells) were assessed under a light/phase-contrast microscope using a 40× objective. To evaluate sperm cell morphology (including the teratozoospermia index reflecting multiple morphological defects per spermatozoon – TZI), native sperm smears were fixed and stained according to the Papanicolaou method (Aqua-Med, Lodz, Poland) and were analyzed under a bright light microscope using a 100× objective oil immersion lens. The concentration of leukocytes (peroxidase-positive cells) was calculated using the Endtz test (LeucoScreen kit, FertiPro N.V., Beernem, Belgium) and assessed in an improved Neubauer hemocytometer.

### Sperm Chromatin Dispersion (SCD) test

To verify sperm nuclear DNA fragmentation, a commercial chromatin dispersion test – a Halosperm G2 ^®^ kit (Halotech DNA, Madrid, Spain) – was applied. The procedure was performed strictly according to the manufacturer’s guidelines and was described in detail in our previous publications. ^33^^–^^36^

**Figure 2.**
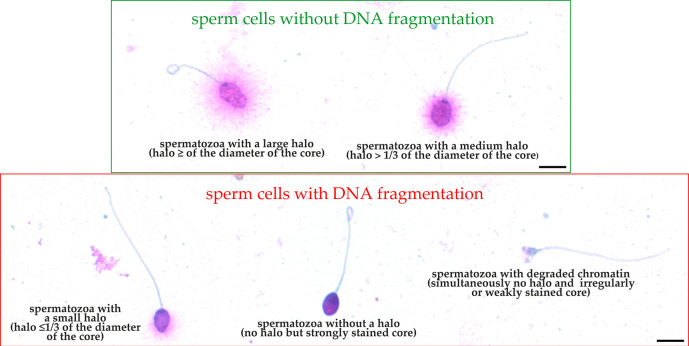
Visualization of sperm chromatin dispersion test (SCD). Micrographs obtained by light microscopy, 100×. Scale bar = 5 μm. Raw micrographs were edited in Corel Photo-Paint 2019 (Corel Corporation, Ottawa, Canada, RRID:SCR_014235). Editing included only: cropping (to center the presented sperm cells), rotation, brightening, contrast enhancing and enlargement.

To calculate the percentage of sperm cells with fragmented DNA, a minimum of 300 sperm cells per sample was counted under the 100x objective of a bright light microscope. According to the manufacturer’s guidelines, the following evaluation criteria were used: (1) sperm cells without nuclear DNA fragmentation (spermatozoa with a large halo – equal to or higher than the diameter of the core of spermatozoa and spermatozoa with a medium-sized halo – >1/3 of the diameter of the core of spermatozoa) and (2) sperm cells with nuclear DNA fragmentation (spermatozoa with a small halo – ≤1/3 of the diameter of the core of spermatozoa and spermatozoa without a halo but with a strongly stained core or without a halo and degraded chromatin – sperm cells showing no halo and simultaneously presenting an irregularly or weakly stained core) ( Figure 2). The results of the SCD test (SDF) are presented as the sum of spermatozoa with nuclear DNA fragmentation divided by the total number of assessed sperm cells and multiplied by 100%.

### Hormone profile of infertile subjects

To assess the panel of basic hormones influencing male fertility, potential vein blood was collected from participants in the morning (7.30–09.00), and the following hormones presented in Table 1 were measured. The hormone levels were determined by the electrochemiluminescence method (ECLIA) using the Cobas e801 analytical unit (Roche Diagnostics GmbH, Mannheim, Germany). The ECLIA method is based on the binding of biotinylated monoclonal-specific antibodies directed against the measured hormones and specific monoclonal antibodies labeled with a complex containing ruthenium metal (sandwich complex). In the next step, streptavidin-bound microparticles were used to bind to biotinylated antibodies directed against the measured hormones. Unbound substances were removed. The bound microparticles were magnetically trapped on the electrode surface. The electrode voltage induced chemiluminescence emission, which was measured by a photomultiplier. The result was determined based on a two-step calibration. All hormonal analyses were assessed strictly in accordance with the manufacturer's instructions.

**Table 1.** Reproductive hormones evaluated in study population.

Hormone	Short description of biological function	Reference value
FSH	FSH levels are considered a marker of impaired spermatogenesis ^8^^,^^10^^,^^11^^,^^105^^,^^106^	1.5–12.4 μIU/mL
LH	LH levels are helpful for differentiating primary (testicular) hypogonadism from secondary (nontesticular) hypogonadism ^10^^,^^11^^,^^54^^,^^106^^,^^107^	1.7–8.6 uIU/mL
total T	Total T levels influences spermatogenesis through Sertoli cells ^10^^,^^11^^,^^108^	8.0–41.7 nmol/L
PRL	PRL levels may result in decreased libido and erectile function ^10^^,^^55^	4.04–15.02 ng/mL
TSH	Both hyperthyroidism and hypothyroidism can negatively affect spermatogenesis ^10^^,^^54^^,^^56^	0.27–4.2 μIU/mL

FSH – follicle-stimulating hormone, LH – luteinizing hormone, PRL – prolactin, TSH – thyroid-stimulating hormone, total T – total testosterone.

### Ultrasonography of the scrotum

To verify testicular volume, USG of the scrotum (ultrasound system Z-5 with a 75L38EA linear head; frequency range of 5–10 MHz, Mindray, Shenzhen, China) was performed by a senior urologist. The measurements were calculated using the following formula: length × width × height × 0.71. Furthermore, they were expressed in mL. According to the most commonly accepted criterion in clinical practice, the hypotrophic gonad was considered when the volume of the testis was less than 12 mL. ^37^ Additionally, the homogeneity and echogenicity of the gonadal parenchyma as well as the presence of possible focal lesions and microcalcifications were assessed.

### Statistical analysis

Because the Shapiro–Wilk test showed that the data were not normally distributed, a nonparametric Mann–Whitney U test and Kruskal–Wallis test were applied to compare quantitative variables between two or more studied groups, respectively. Quantitative variables are expressed as the median with the range and mean ± standard deviation (SD). Additionally, to verify the relationships between study parameters, the Spearman’s rank (r _s_) correlation coefficient was calculated ( Figure 1). To interpret the strength of dependence between parameters, the following levels of correlation were presumed: <0.2 – lack of linear dependence (regardless of the p value), 0.2–0.4 – weak dependence, >0.4–0.7 – moderate dependence, >0.7–0.9 – strong dependence and >0.9 very strong dependence. For all statistical analyses, a p value < 0.05 was considered significant. Data analysis was performed using Statistica version 13.3 (StatSoft, Krakow, Poland, RRID:SCR_014213) and MedCalc version 18.2.1 (MedCalc Software, Ostend, Belgium, RRID:SCR_015044). Open source statistical software which can be used in the study – GNU PSPP.

## Results

### Seminological characteristics of study population

Of 130 obtained semen samples, 26 were classified as normozoospermia (total sperm count ≥39 × 10 ^6^ cells, sperm progressive motility ≥32%, normal sperm morphology ≥4%), 36 as teratozoospermia (abnormal sperm morphology), 36 as oligoasthenoteratozoospermia (simultaneously abnormal total sperm count, progressive motility and morphology), 19 as oligoteratozoospermia (simultaneously abnormal total sperm count and morphology), nine as asthenoteratozoospermia (simultaneously abnormal progressive sperm motility and morphology), three as oligozoospermia (abnormal sperm total count) and one as asthenozoospermia (abnormal sperm progressive motility). Moreover, 51 men had abnormal levels of at least one of the assessed reproductive hormones (FSH, LH, PRL, total T, TSH), and 37 men had an abnormal volume of at least one testis (<12 mL). The descriptive statistics of the investigated parameters are provided in Table 2.

**Table 2.** Descriptive statistics of study parameters in the infertile group of men (n=130).

Study parameters	median (range) mean ± SD
Age (y)	33.00 (23.00–51.00) 33.77 ± 5.28
LTV (mL)	n = 128 15.00 (2.40–25.00) 14.99 ± 4.81
RTV (mL)	n = 128 15.00 (4.30–25.00) 15.50 ± 4.95
FSH (mIU/mL)	n = 128 6.33 (1.17–21.35) 6.84 ± 3.90
LH (mIU/mL)	5.51 (1.21–17.10) 5.66 ± 2.63
total T (nmol/L)	16.60 (3.37–39.48) 17.53 ± 6.22
PRL (ng/mL)	13.22 (5.55–46.56) 14.25 ± 5.90
TSH (μU/mL)	n = 129 1.78 (0.63–14.40) 2.17 ± 1.50
Semen volume (mL)	3.00 (0.50–8.50) 3.34 ± 1.63
Sperm concentration (×10 ^6^/mL)	13.82 (0.40–251.00) 21.60 ± 28.54
Total number of spermatozoa (×10 ^6^)	42.70 (0.84–426.70) 67.76 ± 79.57
Morphologically normal spermatozoa (%)	1.00 (0.00–11.00) 1.80 ± 2.49
TZI	1.75 (1.34–2.50) 1.80 ± 0.29
Sperm progressive motility (%)	44.00 (0.00–87.00) 43.22 ± 22.25
Sperm nonprogressive motility (%)	5.00 (0.00–29.00) 5.56 ± 4.40
Total sperm motility (%)	50.00 (1.00–98.00) 48.79 ± 22.32
Eosin-negative spermatozoa – live cells (%)	76.50 (30.00–96.00) 74.33 ± 13.29
HOS test-positive spermatozoa – live cells (%)	n = 107 78.00 (26.00–92.00) 75.60 ± 10.51
Peroxidase-positive cells (%)	0.25 (0.00–10.25) 0.66 ± 1.32
SDF (%)	20.00 (3.00–58.00) 22.06 ± 12.04

FSH – follicle-stimulating hormone, HOS test – hypo-osmotic swelling test, LH – luteinizing hormone, LTV – left testis volume, SDF – sperm DNA fragmentation, PRL – prolactin, RTV – right testis volume, TSH – thyroid-stimulating hormone, total T – total testosterone, TZI – teratozoospermia index. n – number of subjects, SD – standard deviation.

### Comparison of study parameters between infertile subjects with an abnormal volume of at least one testis (<12 mL) and subjects with a normal volume of testes (each ≥12 mL)

Compared groups did not differ in age. The subjects from the group with abnormal testicular volume (n = 37) had significantly higher levels of FSH than the reference group (n = 91) (median: 8.05 mIU/mL vs. 5.29 mIU/mL), whereas the levels of other study hormones (LH, PRL, total T, TSH) did not differ significantly ( Table 3). On the other hand, in case of seminological parameters, patients with decreased testicular volume had a significantly reduced sperm concentration (medians: 6.25 × 10 ^6^ cells/mL vs. 19.00 ×10 ^6^ cells/mL), total sperm count (medians: 15.35 × 10 ^6^ cells vs. 60.12 × 10 ^6^ cells), sperm morphology (medians: 0.00% vs. 1.00%), progressive motility (medians: 37.00% vs. 51.00%), total motility (medians: 43.50% vs. 57.00%) and vitality – eosin-negative sperm cells (medians: 73.00% vs. 79.00%) and hypoosmotic (HOS) test-positive sperm cells (medians: 73.50% vs. 78.00%). It should be highlighted that in the group of men with abnormal testicular volume, a significantly higher percentage of SDF was found (medians: 27.00% vs. 17.00%) ( Table 4).

**Table 3.** Descriptive statistics and comparison of age, testicular volume and levels of reproductive hormones between infertile subjects with an abnormal volume of at least one testis (<12 mL) and subjects with a normal volume of testes (each ≥12 mL).

Study parameters	Subjects with abnormal volume of at least one testis (<12 mL) n = 37	Subjects with normal volume of testes (each ≥12 mL) n = 91	*p*
**median (range) mean ± SD**			
Age (y)	33.00 (23.00–49.00) 33.34 ± 4.89	33.00 (22.00–48.00) 33.60 ± 5.06	NS
LTV (mL)	10.00 (2.40–18.00) 9.81 ± 3.27	16.00 (12.00–25.00) 17.10 ± 3.58	**<0.000000**
RTV (mL)	10.00 (4.30–24.00) 10.82 ± 4.10	16.70 (9.00–25.00) 17.40 ± 3.90	**<0.000000**
FSH (mIU/mL)	8.05 (2.10–21.35) 8.66 ± 4.23	n = 90 5.29 (1.17–17.00) 6.05 ± 3.30	**0.002677**
LH (mIU/mL)	5.86 (2.30–17.10) 6.37 ± 3.32	5.49 (1.21–11.20) 5.41 ± 2.22	NS
total T (nmol/L)	16.12 (4.16–27.07) 4.76 ± 1.62	17.75 (3.37–39.48) 5.18 ± 1.87	NS
PRL (ng/mL)	13.12 (6.80–25.52) 13.84 ± 5.28	13.20 (5.55–46.56) 14.39 ± 6.18	NS
TSH (μU/mL)	1.61 (0.63–14.40) 2.13 ± 2.21	n = 90 2.01 (0.71–6.70) 2.19 ± 1.09	NS

FSH – follicle-stimulating hormone, LH – luteinizing hormone, LTV – left testis volume, PRL – prolactin, RTV – right testis volume, TSH – thyroid-stimulating hormone, total T – total testosterone. n – number of subjects, NS – no statistical significance, SD – standard deviation. Statistical significance in the Mann–Whitney U test was reached when p < 0.05.

**Table 4.** Descriptive statistics and comparison of semen parameters between infertile subjects with an abnormal volume of at least one testis (<12 mL) and subjects with a normal volume of testes (each ≥12 mL).

Study parameters	Subjects with abnormal volume of at least one testis (<12 mL) n = 37	Subjects with normal volume of testes (each ≥12 mL) n = 91	*p*
**median (range) mean ± SD**			
Semen volume (mL)	3.00 (0.75–6.50) 3.04 ± 1.25	3.00 (0.50–8.50) 3.48 ± 1.76	NS
Sperm concentration (×10 ^6^/mL)	6.25 (0.40–57.25) 11.26 ± 12.63	19.00 (0.45–251.00) 26.05 ± 32.17	**0.000111**
Total number of spermatozoa (×10 ^6^)	15.35 (0.88–169.00) 33.28 ± 39.25	60.12 (0.84–426.70) 82.64 ± 87.72	**0.000046**
Morphologically normal spermatozoa (%)	0.00 (0.00–10.00) 1.05 ± 1.95	1.00 (0.00–11.00) 2.14 ± 2.63	**0.028614**
TZI	1.71 (1.36–2.50) 1.79 ± 0.29	1.75 (1.34–2.48) 1.81 ± 0.28	NS
Sperm progressive motility (%)	37.00 (0.00–87.00) 37.36 ± 20.47	51.00 (2.00–86.00) 45.98 ± 22.49	**0.032661**
Sperm nonprogressive motility (%)	5.00 (0.00–18.00) 5.50 ± 3.98	5.00 (0.00–29.00) 5.63 ± 4.59	NS
Total sperm motility (%)	43.50 (1.00–90.00) 42.86 ± 20.70	57.00 (4.00–88.00) 51.62 ± 22.42	**0.026757**
Eosin-negative spermatozoa –live cells (%)	73.00 (41.00–95.00) 70.07 ± 13.08	79.00 (30.00–96.00) 76.16 ± 13.10	**0.003811**
HOS test-positive spermatozoa – live cells (%)	n = 26 73.50 (54.00–88.00) 72.38 ± 9.60	n = 80 78.00 (26.00–92.00) 76.73 ± 10.66	**0.033568**
Peroxidase-positive cells (%)	0.25 (0.00–10.25) 0.65 ± 1.66	0.25 (0.00–9.50) 0.66 ± 1.17	NS
SDF (%)	27.00 (3.00–58.00) 29.00 ± 14.30	17.00 (4.00–46.00) 19.16 ± 9.75	**0.000127**

HOS test – hypo-osmotic swelling test, SDF – sperm DNA fragmentation, TZI – teratozoospermia index. n – number of subjects, NS – no statistical significance, SD – standard deviation. Statistical significance in the Mann–Whitney U test was reached when p < 0.05.

### Comparison of study parameters between infertile subjects with abnormal levels of at least one of the assessed hormones and subjects with normal hormonal profiles

There were no significant differences in age between the compared groups, whereas higher levels of FSH, LH, PRL and TSH were noted in infertile men with abnormal hormonal profiles (n = 51) than in infertile men with normal hormonal profiles (n = 79). Unexpectedly, the study groups did not differ in the level of total T ( Table 5). Regarding the semen parameters, infertile men with hormonal disorders had significantly lower total sperm count (medians: 30.25 × 10 ^6^ cells vs. 54.00 × 10 ^6^ cells), sperm morphology (medians: 0.00% vs. 1.00%), progressive motility (medians: 35.00% vs. 50.00%) and total motility (medians: 41.00% vs. 57.00%). Furthermore, the percentage of SDF was increased in the group with hormonal abnormalities, but the difference was not statistically significant (medians: 22.00% vs. 18.00%). Additionally, the compared groups did not differ in testicular volume ( Table 6).

**Table 5.** Descriptive statistics and comparison of age, testicular volume and levels of reproductive hormones between infertile subjects with abnormal hormone profiles (abnormal levels of at least one evaluated hormone) and subjects with normal hormone profiles.

Study parameters	Subjects with abnormal hormone profile n = 51	Subjects with normal hormone profile n = 79	*p*
**median (range) mean ± SD**			
Age (y)	33.00 (25.00–47.00) 33.43 ± 4.45	33.00 (22.00–49.00) 33.48 ± 5.44	NS
LTV (mL)	n = 49 14.00 (2.40–23.00) 13.98 ± 5.02	15.40 (6.00–25.00) 15.62 ± 4.59	NS
RTV (mL)	n = 50 14.00 (4.30–24.00) 14.61 ± 4.98	n = 78 15.00 (6.80–25.00) 16.06 ± 4.78	NS
FSH (mIU/mL)	7.70 (1.17–21.35) 7.90 ± 4.23	n = 78 5.40 (1.80–17.87) 6.16 ± 3.53	**0.011483**
LH (mIU/mL)	6.82 (1.45–17.10) 6.94 ± 3.07	4.49 (1.21–8.55) 4.83 ± 1.91	**0.000024**
total T (nmol/L)	15.60 (3.37–28.77) 16.08 ± 6.37	17.95 (8.32–39.48) 5.31 ± 1.74	NS
PRL (ng/mL)	19.10 (6.42–46.56) 18.85 ± 6.35	11.79 (5.55–17.90) 11.29 ± 3.00	**< 0.000000**
TSH (μU/mL)	n = 50 2.17 (0.63–14.40) 2.49 ± 1.98	1.65 (0.70–6.70) 1.97 ± 1.05	**0.028703**

FSH – follicle-stimulating hormone, LH – luteinizing hormone, LTV – left testis volume, PRL – prolactin, RTV – right testis volume, TSH – thyroid-stimulating hormone, total T – total testosterone. n – number of subjects, NS – no statistical significance, SD – standard deviation. Statistical significance in the Mann–Whitney U test was reached when p < 0.05.

**Table 6.** Descriptive statistics and comparison of semen parameters between infertile subjects with abnormal hormone profiles (abnormal levels of at least one evaluated hormone) and subjects with normal hormone profiles.

Study parameters	Subjects with abnormal hormone profile n = 51	Subjects with normal hormone profile n = 79	*p*
**median (range) mean ± SD**			
Semen volume (mL)	3.00 (0.75–7.90) 3.07 ± 1.49	3.50 (0.50–8.50) 3.52 ± 1.70	NS
Sperm concentration (×10 ^6^/mL)	12.87 (0.40–118.50) 17.42 ± 20.70	15.62 (0.45–251.00) 24.31 ± 32.46	NS
Total number of spermatozoa (×10 ^6^)	30.25 (0.88–412.77) 52.53 ± 69.77	54.00 (0.84–426.70) 77.60 ± 84.27	**0.018847**
Morphologically normal spermatozoa (%)	0.00 (0.00–8.00) 1.17 ± 1.95	1.00 (0.00–11.00) 2.21 ± 2.72	**0.013886**
TZI	1.75 (1.35–2.50) 1.82 ± 0.32	1.75 (1.34–2.45) 1.79 ± 0.27	NS
Sperm progressive motility (%)	35.00 (0.00–86.00) 37.19 ± 21.44	50.00 (1.00–87.00) 47.11 ± 22.03	**0.011304**
Sperm nonprogressive motility (%)	5.00 (0.00–18.00) 4.88 ± 3.19	5.00 (0.00–29.00) 6.01 ± 4.99	NS
Total sperm motility (%)	41.00 (1.00–88.00) 42.07 ± 21.56	57.00 (2.00–90.00) 53.12 ± 21.84	**0.005652**
Eosin-negative spermatozoa – live cells (%)	74.00 (44.00–96.00) 73.47 ± 11.54	79.00 (30.00–95.00) 74.88 ± 14.35	NS
HOS test-positive spermatozoa – live cells (%)	n = 41 76.00 (54.00–91.00) 75.12 ± 8.66	n = 66 78.50 (26.00–92.00) 75.90 ± 11.56	NS
Peroxidase-positive cells (%)	0.25 (0.00–10.25) 0.94 ± 2.00	0.25 (0.00–2.15) 0.48 ± 0.51	NS
SDF (%)	22.00 (7.00–58.00) 23.54 ± 12.05	18.00 (3.00–54.00) 21.10 ± 12.02	NS

HOS test – hypo-osmotic swelling test, SDF – sperm DNA fragmentation, TZI – teratozoospermia index. n – number of subjects, NS – no statistical significance, SD – standard deviation. Statistical significance in the Mann–Whitney U test was reached when p < 0.05.

### Comparison of study parameters between infertile subjects with SDF >30%, >15–30% and ≤15%

Based on the publications of other authors, ^16^^,^^19^^,^^22^^,^^23^^,^^25^^,^^26^^,^^29^^,^^31^ the study group was divided into three subgroups: 1) with a high level of sperm nuclear DNA damage (SDF >30%, low fertility potential), 2) with a moderate level of sperm nuclear DNA damage (SDF >15–30%, moderate fertility potential) and 3) with a low level of sperm nuclear DNA damage (SDF ≤15%, high fertility potential) ( Tables 7, 8).

**Table 7.** Descriptive statistics and comparison of age, testicular volume and levels of reproductive hormones between infertile subjects with SDF >30%, >15–30% and ≤15%.

Study parameters	Subjects with SDF >30 (1) n = 28	Subjects with SDF >15–30% (2) n = 59	Subjects with SDF ≤15% (3) n = 43	*p 1 vs. 2*	*p 1 vs. 3*	*p 2 vs. 3*
**median (range) mean ± SD**						
Age (y)	31.00 (27.00–51.00) 33.00 ± 5.63	34.00 (25.00–49.00) 34.35 ± 5.90	34.00 (23.00–43.00) 33.48 ± 4.04	NS	NS	NS
LTV (mL)	13.00 (4.00–23.00) 13.48 ± 4.76	n = 57 14.00 (2.40–25.00) 14.55 ± 5.02	16.00 (9.00–25.00) 16.58 ± 4.15	NS	**0.024251**	NS
RTV (mL)	n = 27 12.00 (8.00–23.00) 13.78 ± 4.86	n = 58 15.00 (4.30–25.00) 15.38 ± 5.12	16.00 (8.00–24.00) 16.73 ± 4.52	NS	**0.032806**	NS
FSH (mIU/mL)	6.32 (1.80–14.50) 6.60 ± 3.55	6.70 (1.93–21.35) 7.51 ± 4.49	n = 42 5.44 (1.17–13.40) 6.06 ± 3.06	NS	NS	NS
LH (mIU/mL)	5.07 (2.10–9.30) 5.45 ± 2.47	5.77 (1.45–17.10) 5.93 ± 3.09	5.20 (1.21–11.20) 5.43 ± 2.01	NS	NS	NS
total T (nmol/L)	18.16 (4.16–28.08) 5.10 ± 1.51	16.19 (3.37–37.20) 17.05 ± 6.76	16.12 (8.66–39.48) 18.09 ± 6.10	NS	NS	NS
PRL (ng/mL)	11.89 (6.42–25.52) 13.89 ± 5.48	14.16 (6.42–28.30) 14.68 ± 4.95	12.14 (5.55–46.56) 13.91 ± 7.30	NS	NS	NS
TSH (μU/mL)	2.01 (0.63–14.40) 2.48 ± 2.52	n = 58 1.73 (0.84–6.70) 2.07 ± 1.06	1.68 (0.71–5.48) 2.11 ± 1.07	NS	NS	NS

FSH – follicle-stimulating hormone, LH – luteinizing hormone, LTV – left testis volume, PRL – prolactin, RTV – right testis volume, TSH – thyroid-stimulating hormone, total T – total testosterone. n – number of subjects, NS – no statistical significance, SD – standard deviation. Statistical significance in the Kruskal–Wallis test was reached when p < 0.05.

**Table 8.** Descriptive statistics and comparison of semen parameters between infertile subjects with SDF >30%, >15–30% and ≤15%.

Study parameters	Subjects with SDF >30 (1) n = 28	Subjects with SDF >15–30% (2) n = 59	Subjects with SDF ≤15% (3) n = 43	*p 1 vs. 2*	*p 1 vs. 3*	*p 2 vs. 3*
**median (range) mean ± SD**						
Semen volume (mL)	3.50 (1.50–8.50) 3.66 ± 1.78	3.00 (0.50–8.00) 3.06 ± 1.47	3.00 (1.50–8.00) 3.52 ± 1.72	NS	NS	NS
Sperm concentration (×10 ^6^/mL)	5.65 (0.40–60.25) 10.90 ± 13.50	13.04 (0.80–104.50) 20.40 ± 21.46	21.75 (2.50–251.00) 30.22 ± 39.93	NS	**0.000175**	NS
Total number of spermatozoa (×10 ^6^)	20.02 (0.88–221.00) 42.42 ± 59.41	41.25 (0.84–365.75) 63.75 ± 78.07	70.76 (8.10–426.70) 89.77 ± 88.40	NS	**0.000841**	**0.025102**
Morphologically normal spermatozoa (%)	0.00 (0.00–3.00) 0.46 ± 0.83	0.00 (0.00–8.00) 1.20 ± 1.74	3.00 (0.00–11.00) 3.51 ± 3.11	NS	**0.000011**	**0.000497**
TZI	1.79 (1.36–2.48) 1.82 ± 0.26	1.69 (1.35–2.50) 1.82 ± 0.33	1.75 (1.34–2.30) 1.77 ± 0.24	NS	NS	NS
Sperm progressive motility (%)	26.00 (6.00–67.00) 28.82 ± 16.00	40.00 (0.00–70.00) 38.54 ± 19.72	63.00 (15.00–87.00) 59.02 ± 19.86	NS	**<0.000001**	**0.000016**
Sperm nonprogressive motility (%)	4.50 (0.00–10.00) 4.39 ± 2.97	6.00 (0.00–29.00) 6.74 ± 5.50	4.00 (0.00–13.00) 4.72 ± 2.89	NS	NS	NS
Total sperm motility (%)	31.00 (6.00–71.00) 33.21 ± 17.18	48.00 (1.00–77.00) 45.28 ± 20.01	68.00 (15.00–90.00) 63.74 ± 19.47	NS	**<0.000001**	**0.000103**
Eosin-negative spermatozoa – live cells (%)	67.00 (30.00–86.00) 62.71 ± 15.82	74.00 (40.00–86.00) 73.13 ± 9.49	86.00 (47.00–96.00) 83.53 ± 8.87	NS	**<0.000001**	**<0.000001**
HOS test-positive spermatozoa – live cells (%)	n = 17 67.00 (26.00–84.00) 65.82 ± 13.67	n = 49 72.00 (53.00–85.00) 72.97 ± 7.44	n = 41 83.00 (67.00–92.00) 82.80 ± 7.01	NS	**<0.000001**	**0.000001**
Peroxidase-positive cells (%)	0.25 (0.00–10.25) 0.81 ± 1.90	0.25 (0.00–9.50) 0.76 ± 1.41	0.25 (0.00–2.00) 0.41 ± 0.51	NS	NS	NS

HOS test – hypo-osmotic swelling test, SDF – sperm DNA fragmentation, TZI – teratozoospermia index. n – number of subjects, NS – no statistical significance, SD – standard deviation. Statistical significance in the Kruskal–Wallis test was reached when p < 0.05.

Statistical analysis revealed some significant differences between men with SDF >30% (n = 28) and men with SDF ≤15% (n = 43). The first group had significantly lower left testis volume (medians: 13.00 mL vs. 16.00 mL) and right testis volume (medians: 12.00 mL vs. 16.00 mL), sperm concentration (medians: 5.65 ×10 ^6^ cells/mL vs. 21.75 ×10 ^6^ cells/mL), total sperm count (medians: 20.02 ×10 ^6^ cells vs. 70.76 ×10 ^6^ cells), sperm morphology (medians: 0.00% vs. 3.00%), progressive motility (medians: 26.00% vs. 63.00%), total motility (medians: 33.00% vs. 68.00%) and vitality – eosin-negative sperm cells (medians: 67.00% vs. 86.00%) and HOS test-positive sperm cells (medians: 67.00% vs. 83.00%) ( Tables 7, 8).

In addition, in contrast to men with SDF ≤15%, infertile men with SDF >15–30% (n = 59) had a significantly lower total sperm count (medians: 41.25 × 10 ^6^ cells vs. 70.76 × 10 ^6^ cells), sperm morphology (medians: 0.00% vs. 3.00%), progressive motility (medians: 40.00% vs. 63.00%), total motility (medians: 48.00% vs. 68.00%) and sperm vitality – eosin-negative sperm cells (medians: 74.00% vs. 86.00%) and HOS test-positive sperm cells (medians: 72.00% vs. 83.00%) ( Table 7).

On the other hand, we did not observe any significant differences between men with SDF >30% and men with SDF >15–30% in any study parameters. Additionally, in the case of age, hormone levels (FSH, LH, PRL, total T, TSH), TZI index, sperm nonprogressive motility and concentration of peroxidase-positive cells in semen, no significant differences between the compared three groups were recorded ( Tables 7, 8).

### Spearman’s rank correlations between the study parameters

*Correlations between SDF, male age, basic semen parameters, testicular volume and hormone levels*

Analysis of the Spearman’s rank correlation coefficient showed a linear relationship between SDF and selected parameters. SDF was negatively correlated with sperm concentration (r _s_ = –0.3461; weak dependence), total sperm count (r _s_ = –0.3343; weak dependence), sperm morphology (r _s_ = –0.4482; moderate dependence), progressive motility (r _s_ = –0.5476; moderate dependence), total motility (r _s_ = –0.5374; moderate dependence) and vitality – eosin-negative sperm cells (r _s_ = –0.6389; moderate dependence) and HOS test-positive sperm cells (r _s_ = –0.5811; moderate dependence). In turn, there were no significant correlations between SDF and age, ejaculate volume, nonprogressive sperm motility or peroxidase-positive cell concentration. Moreover, a negative correlation between SDF and the volume of the left testis was found (r _s_ = –0.2055; weak dependence), whereas there were no significant correlations between SDF and the volume of the right testis or study hormone levels ( Tables 9, 10).

**Table 9.** Spearman's rank correlation coefficient (r _s_) between age, testicular volume and levels of reproductive hormones.

Study parameters	LTV (mL)	RTV (mL)	FSH (mIU/mL)	LH (mIU/mL)	total T (nmol/L)	PRL (ng/mL)	TSH (μU/mL)	SDF (%)
Age (y)	n = 128 r _s_ = -0.0320 NS	n = 128 r _s_ = -0.0767 NS	n = 129 r _s_ = 0.1698 NS	r _s_ = 0.1203 NS	r _s_ = 0.0657 NS	r _s_ = -0.0599 NS	n = 129 r _s_ = -0.0398 NS	r _s_ = -0.0783 NS
LTV (mL)	-	n = 127 **r**_**s**_ **= 0.8259** p < 0.000001	n = 127 **r**_**s**_ **= -0.2491** p = 0.004745	n = 128 r _s_ = -0.1529 NS	n = 128 r _s_ = 0.1225 NS	n = 128 r _s_ = -0.0195 NS	n = 127 r _s_ = 0.0832 NS	n = 128 **r**_**s**_ **= -0.2055** p = 0.019947
RTV (mL)	n = 127 **r**_**s**_ **= 0.8259** p < 0.000001	-	n = 127 **r**_**s**_ **= -0.2402** p = 0.006525	n = 128 r _s_ = -0.1596 NS	n = 128 r _s_ = 0.1418 NS	n = 128 r _s_ = -0.0759 NS	n = 127 r _s_ = 0.0564 NS	n = 128 r _s_ = -0.1666 NS
FSH (mIU/mL)	n = 127 **r**_**s**_ **= -0.2491** p = 0.004745	n = 127 **r**_**s**_ **= -0.2402** p = 0.006525	-	n = 129 **r**_**s**_ **= 0.5018** p < 0.000001	n = 129 r _s_ = 0.0524 NS	n = 129 r _s_ = 0.0720 NS	n = 128 r _s_ = 0.0533 NS	n = 129 r _s_ = 0.0459 NS
LH (mIU/mL)	n = 128 r _s_ = -0.1529 NS	n = 128 r _s_ = -0.1596 NS	n = 129 **r**_**s**_ **= 0.5018** p < 0.000001	-	r _s_ = 0.0568 NS	r _s_ = 0.1673 NS	n = 129 r _s_ = 0.0874 NS	r _s_ = 0.0029 NS
total T (nmol/L)	n = 128 r _s_ = 0.1225 NS	n = 128 r _s_ = 0.1418 NS	n = 129 r _s_ = 0.0524 NS	r _s_ = = 0.0568 NS	-	r _s_ = -0.0703 NS	n = 129 r _s_ = --0.0892 NS	r _s_ = 0.0102 NS
PRL (ng/mL)	n = 128 r _s_ = -0.0195 NS	n = 128 r _s_ = -0.0759 NS	n = 129 r _s_ = 0.0720 NS	r _s_ = 0.1673 NS	r _s_ = -0.0703 NS	-	n = 128 r _s_ = 0.1514 NS	r _s_ = 0.0802 NS
TSH (μU/mL)	n = 127 r _s_ = 0.0832 NS	n = 127 r _s_ = 0.0564 NS	n = 128 r _s_ = 0.0533 NS	n = 129 r _s_ = 0.0874 NS	n = 129 r _s_ = -0.0892 NS	n = 129 r _s_ = 0.1514 NS	-	n = 129 r _s_ = 0.0321 NS

FSH – follicle-stimulating hormone, LH – luteinizing hormone, PRL – prolactin, RTV – right testis volume, TSH – thyroid-stimulating hormone, total T – total testosterone. n – number of subjects, NS – no statistical significance, SD – standard deviation. Statistical significance was reached when *p* < 0.05.

**Table 10.** Spearman's rank correlation coefficient (r _s_) between testicular volume, levels of reproductive hormones and semen parameters.

Study parameters	LTV (mL)	RTV (mL)	FSH (mIU/mL)	LH (mIU/mL)	total T (nmol/L)	PRL (ng/mL)	TSH (μU/mL)	SDF (%)
Semen volume (mL)	n = 128 r _s_ = 0.0376 NS	n = 128 r _s_ = -0.0524 NS	n = 129 r _s_ = -0.0102 NS	r _s_ = -0.1285 NS	r _s_ = -0.0994 NS	r _s_ = -0.1039 NS	n = 129 r _s_ = -0.0278 NS	r _s_ = -0.0265 NS
Sperm concentration (×10 ^6^/mL)	n = 128 **r**_**s**_ **= 0.4345** p < 0.000001	n = 128 **r**_**s**_ **= 0.4019** p = 0.000003	n = 129 r _s_ = -0.1720 NS	**r**_**s**_ **= -0.2205** p = 0.011703	r _s_ = -0.0564 NS	r _s_ = -0.0760 NS	n = 129 r _s_ = -0.0152 NS	**r**_**s**_ **= -0.3461** p = 0.000055
Total number of spermatozoa (×10 ^6^)	n = 128 **r**_**s**_ **= 0.4191** p < 0.000001	n = 128 **r**_**s**_ **= 0.3452** p = 0.000066	n = 129 r _s_ = -0.1850 p = 0.035873	**r**_**s**_ **= -0.2350** p = 0.007108	r _s_ = -0.1067 NS	r _s_ = -0.1011 NS	n = 129 r _s_ = -0.0220 NS	**r**_**s**_ **= -0.3343** p = 0.000101
Morphologically normal spermatozoa (%)	n = 128 r _s_ = 0.1634 NS	n = 128 r _s_ = 0.1579 NS	n = 129 r _s_ = 0.0063 NS	r _s_ = -0.0681 NS	r _s_ = -0.0269 NS	r _s_ = -0.1368 NS	n = 129 r _s_ = -0.0006 NS	**r**_**s**_ **= -0.4482** p < 0.000001
TZI	n = 128 r _s_ = 0.0062 NS	n = 128 r _s_ = 0.0549 NS	n = 129 r _s_ = 0.0152 NS	r _s_ = 0.0235 NS	r _s_ = 0.1821 p = 0.037884	r _s_ = 0.0066 NS	n = 129 r _s_ = 0.1334 NS	r _s_ = 0.0525 NS
Sperm progressive motility (%)	n = 128 **r**_**s**_ **= 0.2048** p = 0.020393	n = 128 r _s_ = 0.1313 NS	n = 129 r _s_ = 0.0378 NS	r _s_ = -0.0940 NS	r _s_ = -0.0095 NS	r _s_ = -0.1121 NS	n = 129 r _s_ = -0.1199 NS	**r**_**s**_ **= -0.5476** p < 0.000001
Sperm nonprogressive motility (%)	n = 128 r _s_ = 0.0562 NS	n = 128 r _s_ = -0.0665 NS	n = 129 r _s_ = 0.0030 NS	r _s_ = 0.0059 NS	r _s_ = -0.0287 NS	r _s_ = 0.0462 NS	n = 129 r _s_ = -0.0200 NS	r _s_ = 0.0202 NS
Total sperm motility (%)	n = 128 **r**_**s**_ **= 0.2115** p = 0.016524	n = 128 r _s_ = 0.1236 NS	n = 129 r _s_ = 0.0207 NS	r _s_ = -0.1126 NS	r _s_ = -0.0096 NS	r _s_ = -0.1008 NS	n = 129 r _s_ = -0.1180 NS	**r**_**s**_ **= -0.5374** p < 0.000001
Eosin-negative spermatozoa – live cells (%)	n = 128 r _s_ = 0.1956 p = 0.026909	n = 128 r _s_ = 0.0945 NS	n = 129 r _s_ = -0.0102 NS	r _s_ = -0.0470 NS	r _s_ = 0.1652 NS	r _s_ = -0.0577 NS	n = 129 r _s_ = -0.0300 NS	**r**_**s**_ **= -0.6389** p < 0.000001
HOS test-positive spermatozoa – live cells (%)	n = 105 r _s_ = 0.1904 NS	n = 105 r _s_ = 0.0934 NS	n = 106 r _s_ = -0.0359 NS	n = 107 r _s_ = -0.0147 NS	n = 107 r _s_ = 0.1867 NS	n = 107 r _s_ = -0.0708 NS	n = 106 r _s_ = 0.0016 NS	n = 107 **r**_**s**_ **= -0.5811** p < 0.000001
Peroxidase-positive cells (%)	n = 128 r _s_ = 0.1248 NS	n = 128 r _s_ = 0.1629 NS	n = 129 r _s_ = -0.0553 NS	r _s_ = 0.0387 NS	r _s_ = 0.1138 NS	r _s_ = -0.0247 NS	n = 129 r _s_ = 0.1441 NS	r _s_ = 0.1906 p = 0.026503

FSH – follicle-stimulating hormone, HOS test – hypo-osmotic swelling test, LH – luteinizing hormone, LTV – left testis volume, SDF – sperm DNA fragmentation, PRL – prolactin, RTV – right testis volume, TSH –thyroid-stimulating hormone, total T – total testosterone, TZI – teratozoospermia index. n – number of subjects, NS – no statistical significance, SD – standard deviation. Statistical significance was reached when *p* < 0.05.

*Correlations between testicular volume, hormone levels and basic semen parameters*

In the examined group, the left and right testis volumes were negatively correlated with the level of FSH (r _s_ = –0.2491 and r _s_ = –0.2402, respectively; weak dependences) but was not correlated with other hormones (LH, PRL, total T, TSH). Moreover, the volumes of the left and right testes were positively correlated with sperm concentration (r _s_ = 0.4345 and r _s_ = 0.4019, respectively; moderate dependences) and total sperm number (r _s_ = 0.4191 and r _s_ = 0.3452, respectively; moderate and weak dependences). Additionally, positive correlations between left testis volume and sperm progressive motility (r _s_ = 0.2048) as well as total motility (r _s_ = 0.2115; weak dependence) were found. Furthermore, the LH level was negatively correlated with sperm concentration (r _s_ = –0.2205; weak dependence) and total sperm count (r _s_ = –0.2350; weak dependence), but there were no other significant correlations between the levels of assessed hormones and conventional semen parameters ( Tables 9, 10).

The raw data can be found as *Underlying data.* ^109^^,^^110^

## Discussion

### Reduced basic semen parameters can result from impaired spermatogenesis

Based on the obtained data, it can be suggested that the failure to become a biological father could be due to disorders of spermatogenesis manifested by reduced standard seminological parameters. It is worth noting that in our study, the median morphologically normal sperm was only 1%, and as many as 100 out of 130 infertile men had teratozoospermia (isolated or coexisting with other semen disorders). Additionally, studies conducted by other authors confirm the relationship between standard sperm parameters and male fertility. ^38^^–^^40^ Slama *et al*. ^40^ proved a significantly shorter time to pregnancy (TTP) in women whose partners had a higher percentage of sperm with normal morphology. Moreover, it was found that the percentage of morphologically normal sperm was decreased in men from couples with a history of recurrent miscarriage. ^41^^–^^44^ Additionally, morphologically normal sperm cells play an important role not only in the case of natural conception but also in medically assisted conception (IUI, fertilization *in vitro*), ^45^ and it has been shown that sperm morphology may also influence embryo development. ^43^ On the other hand, reproductive success might be achieved even when morphologically normal sperm cells are not observed in the semen. Shabtaie *et al*. ^46^ emphasize that in the case of only abnormal sperm morphology (assuming no female infertility factor), first-line therapy should not assist ART without undertaking a sufficiently long attempt at natural conception. Therefore, opinions about the predictive value of sperm morphological assessment for both natural conception and medically assisted conception are controversial. ^46^^–^^49^

Additionally, in our study, 46 cases of asthenozoospermia (isolated or coexisting) and 58 cases of oligozoospermia (isolated or coexisting) were observed. Many authors confirm that progressive motility is one of the most important parameters influencing reproductive success both in terms of natural conception and medically assisted conception. ^38^^,^^50^^,^^51^ Furthermore, Lotti *et al.* ^39^ revealed a negative correlation between sperm vitality and TTP. Also, analyzing a large group of infertile men and men from the control group (candidates to be sperm donors), Li *et al*. ^43^ showed that in the first group, there were significantly more men with azoospermia, asthenozoospermia and oligoasthenozoospermia, whereas surprisingly isolated oligoozospermia were detected more often in men from the control group. Some authors ^52^ even suggest greater clinical implications of the total sperm count in relation to the sperm concentration.

However, it should be emphasized that low standard seminological parameters are not always synonymous with infertility status. Not all authors ^53^ recognize the arbitrary division of men into fertile and infertile groups based only on the basic semen characteristics according to the WHO. ^32^ Therefore, except for azoospermia, necrozoospermia, and globo- and macrozoospermia, it is difficult to determine male fertility potential considering only standard seminological parameters. This thesis is also confirmed by our previous studies, in which 19 cases of reduced basic semen parameters were found in a group of men with proven fertility (n = 64). ^33^ Therefore, in this research, the standard seminological assessment was not only one criterion for qualifying a man as infertile. The patient's infertility was established by an interview indicating unsuccessful attempts for offspring during one year of regular intercourse without the use of contraception.

### Relationships between testicular volume, hormone levels, basic semen parameters and sperm genome integrity

The results of our research suggest the coexistence of spermatogenesis disorders with a reduced testicle volume and a higher FSH level. It is known that the process of spermatogenesis, reflecting testicular function, depends on a hypothalamic–pituitary–gonadal axis function, in which gonadotropins LH and FSH play a key role in maintaining testosterone biosynthesis and the function of the seminiferous epithelium, respectively. In addition, it has been proven that the function of the male gonad is influenced by thyroid hormones and prolactin. ^10^^,^^54^^–^^57^ Importantly, a significant decrease in testicular volume can be associated with both reduced hormonal activity (lower levels of androgens) and reproductive activity manifested by seminiferous tubule atrophy. ^58^^–^^60^ Therefore, our study included an evaluation of not only standard seminological parameters but also testicular volume and reproductive hormone levels (FSH, LH, PRL, total T, TSH). It should be noted that the selection of the assessed hormones was based on data from the literature. ^11^^,^^61^ Unfortunately, to date, there have been no strict guidelines regarding the hormonal test profile that should be determined in the routine diagnosis of male infertility. On the other hand, the European Academy of Andrology (EAA) in guidelines from 2018 postulates evaluation of total T, FSH and LH in every case of an infertile man with oligoasthenoteratozoospermia (OAT). ^61^ These recommendations are in line with the guidelines of the European Society Urology (EAU) from 2021. ^11^ However, it is believed that the remaining hormonal tests should be performed based on an individual assessment of the patient. The levels of commonly recognized markers of spermatogenesis and Sertoli cell function (FSH, inhibin B) have been most frequently studied in the available literature. ^62^^–^^65^ In addition, the authors of the study also paid attention to the analysis of the levels of SHBG, ^66^ prolactin, ^67^ estradiol, ^66^ TSH, ^68^^–^^70^ cortisol, ^71^^,^^72^ growth hormone (GH) and insulin-like growth factor 1 (IGF-1). ^73^

Based on testicular volume measurement in our study, two groups of infertile participants were distinguished: men with a volume of at least one testis below the norm (<12 mL) and men with a normal volume of both testes. ^74^ Our results showed that infertile men with a reduced volume of at least one testis had a significantly higher FSH level and a lower sperm count, sperm morphology, motility and vitality. Moreover, it should be especially highlighted that we found a significantly increased fragmentation of sperm nuclear DNA in the first group. These results were confirmed by correlation analysis. The testicular volume was negatively correlated with the level of FSH and the SDF value and positively correlated with the number and motility of sperm. Surprisingly, we did not find an association between testicular volume and total T level.

The obtained findings were partially consistent with the data published by other authors. Numerous researchers have reported relationships between testicular volume, conventional semen parameters, gonadotropin and testosterone levels as well as the results of advanced sperm tests (chromatin status, mitochondrial potential, apoptosis). ^57^^,^^60^^,^^75^^–^^78^ The coexistence of reduced standard semen parameters, decreased testosterone levels and testicular volume presented *by Bahk et al.* ^75^ and Condorelli *et al*. ^57^ suggest that the reduction of testicular volume may be associated not only with impaired spermatogenesis but also with decreased hormonal function of male gonads. Condorelli *et al*. ^57^ recommend periodic assessment of testosterone levels for patients with hypotrophic gonads. On the other hand, the obtained results presented by other authors are not always unambiguous. For example, in contrast to our results, Condorelli *et al.* ^57^ revealed a relationship between testicular volume and testosterone levels, but they did not find a correlation between testicular volume and gonadotropin levels.

As mentioned above, we discovered that a group of men with at least one testis volume <12 mL had significantly reduced integrity of the sperm genome. The data could suggest that spermatogenesis disorders coexist with decreased testicular volume and are manifested not only by reduced conventional sperm parameters but also by molecular disorders of sperm chromatin. The relationship between testicular volume and sperm DNA strand brakes was also confirmed by our other findings. The participants with a high level of SDF (>30%) had significantly smaller testes than men with a low level of SDF (≤15%). Moreover, we noted a negative correlation between testicular volume and sperm chromatin fragmentation. Similar results were obtained by other authors who observed a negative correlation between the fragmentation of nuclear sperm DNA (verification using the TUNEL method), its denaturation (verification using acridine orange), sperm chromatin density (verified using propidium iodide) and the volume of testes. ^57^^,^^76^^,^^78^

In the next step of our research, we compared two groups of subjects: men with abnormal levels of at least one of the assessed hormones and men with normal hormonal profiles. The obtained findings provided nonobvious data. We noted that in the first group, sperm count, morphology and motility were reduced, but testicular volume did not differ significantly between the two groups. Moreover, the LH level was negatively correlated with the total sperm count. Additionally, other authors have confirmed the statistical relationship between the level of selected hormones and standard seminological parameters. ^63^^,^^79^^,^^80^ Wei *et al.* ^80^ showed that in patients with OAT, total T was positively correlated with sperm morphology, whereas PRL was correlated with sperm concentration and motility. Moreover, Lu *et al.* ^79^ and Uhler *et al*. ^63^ revealed a negative correlation between FSH level and semen volume, sperm concentration, morphology and motility as well as between LH level and sperm concentration in infertile men or healthy volunteers.

It should be pointed out that we did not find significant differences in the percentage of sperm cells with fragmented DNA between participants with abnormal levels of at least evaluated hormones and those with normal hormonal profiles. This comparative analysis was consistent with Spearman's rank correlation test, which did not show a significant correlation between the SDF value and the level of the assessed hormones. However, other authors’ data indicated statistical relationships between sperm chromatin quality and the hormonal profile ^79^^,^^81^^,^^82^ The coexistence of sperm DNA fragmentation with abnormally high or low levels of gonadotropins was shown in research published by Wdowiak *et al.* ^82^ These results were partially consistent with the studies of Lu *et al.* ^83^ and Smit *et al.,* ^81^ who showed a negative correlation between sperm DNA fragmentation and elevated levels of FSH and LH. In turn, the association between sperm nuclear DNA damage and testosterone level is not always unequivocal. Some researchers Wdowiak *et al.* ^82^ have found a negative correlation between these parameters, whereas others did not confirm these findings. ^81^^,^^83^

The open question is why there was no statistically significant difference in our research in the percentage of SDF between the groups of men differing in the level of at least one of the assessed hormones. There is no doubt that the obtained results could have been influenced by the limited number of infertile men (n = 130) enrolled in our study and the hormonal heterogeneity of the group of men with abnormal levels of at least one of the verified hormones. Disturbances in the level of hormones can be both a factor influencing infertility and a consequence of such a state. In other words, an abnormal hormonal profile can be responsible for reduced semen quality or may be only a secondary effect of pathological processes in testes. In addition, it should be emphasized that there are many potential factors (e.g., obesity, occupational exposure, comorbidities, age, pharmacotherapy, stress) that may affect the interrelationship between spermatogenesis and hormone levels. ^84^^–^^90^

### Sperm genome integrity is a key point for male fertility

It can be assumed that in our investigated group of infertile patients, one of the major factors that limited the ability of male gametes to fertilize was probably an increased level of sperm nuclear DNA fragmentation. It was found that the group of men with SDF >30% had a significantly reduced sperm count, morphology, motility and vitality in comparison to infertile men with a normal SDF rate of ≤15%. Similarly, Erenpreiss *et al*. ^91^ showed that if males had diagnosed astheno- and teratozoospermia, the odds ratio (OR) for >20% DFI or for >30% DFI was 1.9–4.0-fold higher or 2.8–6.2-fold higher, respectively, than in subjects with normal sperm motility and morphology. Additionally, Vinnakota *et al*. ^29^ observed a decrease in sperm motility in participants with SDF >30%. Moreover, we showed that the level of SDF was negatively correlated (Spearman’s rank correlation test) with sperm count, morphology, motility and vitality, and our findings have been confirmed by the research of other authors. ^30^^,^^83^^,^^92^^,^^93^ However, some researchers have not always found a correlation between SDF and basic sperm parameters. ^94^^–^^96^

Importantly, it should be highlighted that in our study, the median SDF was 20%. In fact, according to the manufacturer of the Halosperm G2 ^®^ kit, these results are in the normal range (SDF below 30%). It seems that the threshold of 30% SDF is too high (risk of a false-negative result). In our previous publications, we demonstrated that the median SDF in the group of men with confirmed fertility and/or with high reproductive potential (healthy volunteers with normozoospermia) ranged from 12% to 14%. ^33^^–^^36^ In addition, these studies also showed a significantly satisfactory predictive value of the sperm chromatin dispersion (SCD) test to discriminate males with normal reproductive potential from those with reduced reproductive potential (based on receiver operating characteristic [ROC] analysis). The cut-off value was 18% and 20% SDF. ^34^^–^^36^ Moreover, we obtained a threshold of 18% SDF to distinguish infertile men from fertile men (unpublished data).

These observations were in agreement with other authors who also clearly showed that the level of sperm nuclear DNA fragmentation was correlated with male infertility and that the acceptable threshold for sperm genome fragmentation was not below 30% but rather below 20%. ^4^^,^^16^^,^^21^^,^^27^^–^^29^^,^^91^^,^^97^^–^^102^ For example, Bungum *et al.* ^97^ showed that in the case of subjects with sperm nuclear DNA fragmentation in the range of 0–20%, the chance of spontaneous pregnancy is constant, but an increase in sperm DNA fragmentation >20% is associated with a reduced ability to achieve pregnancy. Moreover, Majzoub *et al.* ^101^ estimated that the mean value of SDF for fertile subjects was 15.68%, whereas in the infertile group, it was 27.60%. In turn, comparing the groups of fertile and infertile men, Wiweko and Utami ^102^ found not only significant differences in the SDF value between the study groups (medians: 19.90% vs. 29.95%, respectively) but also reported that SDF at the cutoff point of 26.1% had a higher diagnostic value. Similar results were presented by Javed *et al*. ^100^ (the SDF at the cutoff point was 24.47%). Moreover, Evenson ^16^ emphasized that the percentage of sperm with damaged chromatin >15–25 could increase the risk of male infertility and that >20–35% spermatozoa with damaged DNA could significantly reduce the chances of becoming pregnant using *in vitro* fertilization. Therefore, based on own research and analysis of the results of other researchers, Evenson ^16^ concluded that when the percentage of sperm with abnormal chromatin status was >20, male fertility was decreased, and *in vitro* fertilization as first-line therapy should be considered. These conclusions were also confirmed by Giwercman *et al.,* ^103^ who performed a comparison of ORs for the occurrence of infertility depending on the percentage of DFI. The authors showed that in the group of men with DFI 10–20%, the risk of reproductive failure was higher (OR = 2.5) than that in men with DFI <10%. In addition, Giwercman *et al.* ^103^ observed a significant increase in the risk of infertility (OR = 8.4) in men with DFI> 20% compared to men with DFI <10%. Finally, in two most recent publications both Esteves *et al.* ^4^ and Agarwal *et al.* ^21^ reported that cut-off point of 20% sperm cells with fragmented DNA (verified both by SCSA, TUNEL and SCD assay) is the best criterion to discriminate fertile men from infertile.

Additionally, the influence of DFI on the fertilization process has been confirmed. Simon *et al.* ^51^ revealed a higher risk (OR = 9.5) of a low percentage of fertilized oocytes (<40% fertilized oocytes) when men had DFI >40% compared to men with DFI ≤40%. Therefore, we can assume that sperm chromatin abnormalities may be accompanied by lowered standard sperm parameters synergistically affecting male fertility.

## Conclusions

Our comprehensive assessment of male infertility factors allowed us to conclude that in the study clinical cases, spermatogenesis disorders coexisted with decreased testicular volume and increased FSH levels. Moreover, they were manifested not only by reduced basic sperm characteristics but also, very importantly, by a high level of sperm nuclear DNA damage, which has great clinical utility both in terms of natural conception and in terms of ART ( Figure 1). Furthermore, our current and previous findings suggest that the cut-off value of 30% SDF given by manufacturer of the Halosperm G2 ^®^ kit seems too high and should be revised/downgraded to 20%, for better prognosis of male fertility. What’s more, clarification of the relationship between standard semen parameters, testicular volume, levels of reproductive hormones, SDF and clinical features might help to develop new personalized strategies for therapeutic interventions. In the case of infertile men, a complete andrological examination including in-depth medical interview, physical examination, standard semen analysis, scrotal ultrasound, assessment of reproductive hormones and integrity of sperm genome is justified. This medical approach is necessary not only due for verification of the causes of infertility but also due to the need to detect serious health disorders that may be life-threatening. For example, it has been proven that infertile men have an increased risk of testicular cancer, which determines the recommendation of periodic ultrasound examinations of the scrotum and gonadal self-examination. ^11^^,^^104^ Therefore, the introduction of a complex diagnosis of male infertility factors is justified and needed.

### Limitations of the study

Some limitations of our study should be addressed. One of the most important factors influencing our results is the limited number of participants. In total, 130 men from couples with confirmed infertility were included to this project. It is known that the most reliable data are obtained from well-designed studies on large cohorts of patients. Due to the limited number of participants in our research, the presented results should be approached critically, and it should be borne in mind that studies conducted on a larger group could provide different results and conclusions. Moreover, the number of compared men in particular groups was not equal, which may affect the obtained statistical differences between groups. In the assessed hormonal profile, we did not include the determinations of some markers which could also be important for assessing the status of male fertility (i.a. inhibin B, SHBG, GH, estradiol, cortisol). Finally, sperm chromatin dispersion (SCD) test was performed to assess SDF. This test is a standardized diagnostic method but often not considered the gold standard for sperm DNA assessment because it does not directly evaluate breaks of DNA.

## Data availability

### Underlying data

Zenodo: Evaluation of selected semen parameters and biomarkers of male infertility – preliminary study. https://doi.org/10.5281/zenodo.6536196. ^109^

This project contains the following underlying data:

- Kups et al. for database.xlsx (raw data)

Zenodo: Evaluation of selected semen parameters and biomarkers of male infertility – preliminary study. https://doi.org/10.5281/zenodo.6538474. ^110^

This project contains the following underlying data:

- Raw microphotographs

### Extended data

Zenodo: Evaluation of selected semen parameters and biomarkers of male infertility – preliminary study. https://doi.org/10.5281/zenodo.6542238. ^111^

This project contains the following extended data:

- Urological and andrological medical interview Michal Kups.pdf (Patient card used during the medical interview)

Data are available under the terms of the Creative Commons Attribution 4.0 International license (CC-BY 4.0).
