# Supplementary material for: Evaluation of selected semen parameters and biomarkers of male infertility – preliminary study
Source: F1000Res. 2022 May 30;11:591. [Version 1] doi: 10.12688/f1000research.121622.1 (PMC10905134; doi:10.12688/f1000research.121622.1)
Supplement: Peer review history

# Reviewer report: Reviewer response for version 1

**DOI:** 10.5256/f1000research.133505.r238146

**Published:** 2024-02-20

**Authors:** Eva Tvrdá

**Affiliations:** 1 Slovak University of Agriculture, Nitra, Slovakia

This is a nicely written and appropriately designed study evaluating and interconnecting standard semen parameters in infertile men.

 While I appreciate papers that turn "back to the basics" and re-evaluate/re-verify the importance of traditional parameters of sperm quality, I am missing the originality of the study. The authors should place more emphasis on the originality and scientific rigour of their experiments as well as provide a solid justification as to why such types of papers on generally well-accepted knowledge are necessary.

 Finally, the title of the article hints evaluation of biomarkers of male infertility. if so, what are the biomarkers and why should these be more of a center focus for the andrologists?

Is the work clearly and accurately presented and does it cite the current literature?

Yes

If applicable, is the statistical analysis and its interpretation appropriate?

Yes

Are all the source data underlying the results available to ensure full reproducibility?

Yes

Is the study design appropriate and is the work technically sound?

Yes

Are the conclusions drawn adequately supported by the results?

Yes

Are sufficient details of methods and analysis provided to allow replication by others?

Yes

Reviewer Expertise:

Sperm quality, oxidative stress, antioxidants, molecular andrology.

I confirm that I have read this submission and believe that I have an appropriate level of expertise to confirm that it is of an acceptable scientific standard, however I have significant reservations, as outlined above.

# Reviewer report: Reviewer response for version 1

**DOI:** 10.5256/f1000research.133505.r238141

**Published:** 2024-02-20

**Authors:** Nafisa Balasinor

**Affiliations:** 1 ICMR-National Institute for Research in Reproductive and Child Health, Parel, India

In the present manuscript the authors have studied the association between standard semen parameters, testicular volume, reproductive hormones profile and the sperm nuclear DNA fragmentation.

 Overall the study is well done and has clinical utility. However, there are few points which needs to be addressed:

 1. How was the sample size of 130 calculated?

 2. Was infertility due to known female factor ruled out?

 3. The authors should mention how they categorized abnormal hormonal levels.

 4. Table 3: volume in the right testis of normal volume group ranged between 9 to 25ml. However, testicular volume of > or equal to12 was taken as normal testicular volume. Please check the data.

 5. Table 5: Men with abnormal hormonal profile group had increase in all hormones except Testosterone. However, the values do not indicate so. Hence need to check if correct statistical tool was used.

 6. The authors need to check testosterone levels in this infertile group as compared to fertile group or normal range.

 7. SDF in men with normal semen and sperm parameters should be analyzed. If SDF is found in this group too than the importance to including SDF in male infertility work out will be evident.

 8. It would be interesting to see the sperm parameters in individuals in which all 3 parameters, namely, Volume, hormone profile and SDF.

Is the work clearly and accurately presented and does it cite the current literature?

Yes

If applicable, is the statistical analysis and its interpretation appropriate?

I cannot comment. A qualified statistician is required.

Are all the source data underlying the results available to ensure full reproducibility?

Yes

Is the study design appropriate and is the work technically sound?

Partly

Are the conclusions drawn adequately supported by the results?

Partly

Are sufficient details of methods and analysis provided to allow replication by others?

Partly

Reviewer Expertise:

Reproductive endocrinology, male fertility and epigenetics

I confirm that I have read this submission and believe that I have an appropriate level of expertise to confirm that it is of an acceptable scientific standard, however I have significant reservations, as outlined above.

# Reviewer report: Reviewer response for version 1

**DOI:** 10.5256/f1000research.133505.r228728

**Published:** 2024-02-01

**Authors:** Abdul S Ansari

**Affiliations:** 1 Department of Zoology, University of Rajasthan, Jaipur, Rajsthan, India

In the present manuscript the author(s) evaluated etiopathogenesis of male infertility by investigation of the relationship between standard semen parameters, testicular volume, levels of reproductive hormones and the fragmentation of sperm nuclear DNA (SDF). A total of 130 subjects, divided into three groups, with at least one abnormal testis volume, minimum low level among any reproductive hormones and with SDF. Results obtained from the study revealed that the subjects with decreased testicular volume and abnormal levels of hormones were observed with decreased basic semen parameters. Abnormal testicular volume also had, respectively, higher percentage of SDF and enhanced levels of FSH. Subjects with high SDF level had low testicular volume and seminal parameters. Authors concluded that spermatogenesis disorders coexisted with decreased testicular volume and increased FSH levels which manifested by reduced seminal characteristics and high sperm nuclear damage. However, the research article contains several lacunae in terms of writing the manuscript, language, presentation of data, etc. Moreover, the title of the article does not match the contents of the article. It correlated several parameters.

**Specific comments:**

**Abstract: **

1. Page 1:  Methods: The participants of the study were not patients, instead they are subjects. Modify the sentence as “Participant subjects (n=130) were divided into”.

**Introduction:**

1. Page 3, Introduction: Write full form of SCSA test followed by its abbreviation SCSA in parentheses.
2.  Page 3, Methods (Study population): The study population , i.e., 130 male infertile subjects is very small. If the number would have been more, it is easy to draw a conclusion.
3.  Page 3, Methods (Study population): Replace “All patients …” with All subjects …”.
4. Page 3, Methods (Study population): How many subjects with azoospermia and cryptozoospermia were excluded? Mention their number.
5. Page 3, Methods (Study population): Replace the phrase “seminal cords” with “spermatic cords”.
6. Page 4, Methods (Conventional semen analysis): Room temperature is not -20 °C. Correct the sentence.
7. Page 4, Methods (Conventional semen analysis): Add “liquefaction time” of semen as a macroscopic semen analysis parameter.
8. Page 4, Methods (Conventional semen analysis): The terminology total sperm count has changed by WHO as “sperm density” (million/ejaculate). Replace the same with new terminology.
9. Page 4, Methods (Conventional semen analysis): The sperm  vitality or viability is carried out the mixing of a well mixed semen sample with eosin-nigrosin stain and and spreaded on a slide and dried. The unstained spermatozoa are regarded as alive/vital, while pink coloured sperms are considered dead. The HOS test is done with the mixing of a semen sample with a hypoosmotic solution and incubated. After incubation spermatozoa with coiled tails are counted, and calculated in percent (%), termed as HOS positive sperms. How vitality indicating both eosin-positive cells and hypoosmotic-reactive cells (HOS test-positive cells can be assessed?
10. Page 4, Methods (Sperm Chromatin Dispersion (SCD) test): The chromatin dispersion test carried out in the study with Halosperm G2 kit is sort of In vitro Nuclear Chromatin Decondensation (NCD) Test of WHO routinely carried out for infertility diagnosis for fertilizing ability. In the test Sperm heads decondensed which results into swelling of heads, appears as, halos.  Whereas, Non-fertilizable sperm heads remain condensed due to defective nuclear chromatin.  Decondensed sperms heads are counted versus condensed sperm heads and represented as percent. I am surprised that why so expensive SCD test was carried out when other several simple tests are available. A detailed comment is required from author(s) on this point.
11. Page 4, Methods (Hormone profile of infertile subjects): Add Inter-assay and Intra-asssay coefficient of variation of each hone assayed.

**Results:**

1. Page 6, Seminological Characteristics of study population:  Replace the word “Seminological” with “Seminal”.
2.  Page 6, Comparison … (each ≥ 12 mL): Write P (significant) value of FSH.

**Discussion:**

1. Page 14, Para 1, Line 5: This is not a thesis, instead a study or investigation. Change the phrase “This thesis” with an appropriate phrase.
2.  Page 14, Para 3, Line 2: Complete the word “norm” as “normal”.
3. The write-up of discussion is too lengthy, reduced the same to half length.
4. **Conclusions:**
5. A brief conclusion of the study carried out similar with Abstract is required.
6. **Data availability:**
7. Replace the word “project” with “research article”.
8. **References:**
9. Reference Nos. 27, 51: Italicize the words “in Vitro”.
10. Reference 59: The title of the reference in upper (capital) case. Change the same as per Instruction to Authors.
11. References 109 to 111 are related to the present research article, thus should be delated from the list of References.
12. **Tables:**
13. Table 1: This table contains all assayed hormones in the study and well described in the text, therefore, should be deleted.
14. Tables 2, 4, 6, 8, 10:  In all these tables data on Sperm nonprogressive motility (%) have been depicted. When data on Sperm progressive motility (%) are presented, there is no use of depicting this. The rest of Sperm progressive motility (%) will obviously related to the Sperm nonprogressive motility (%).
15. The present study contains a total 10 tables. Presentation of data in the present research article merely only in tables is not so impressive and very hard to understand by readers. Prepare histograms or bar diagrams for depiction of data.
16. Table 10: The data of this table should be depicted with Scatter diagrams.

Is the work clearly and accurately presented and does it cite the current literature?

Yes

If applicable, is the statistical analysis and its interpretation appropriate?

I cannot comment. A qualified statistician is required.

Are all the source data underlying the results available to ensure full reproducibility?

Yes

Is the study design appropriate and is the work technically sound?

Yes

Are the conclusions drawn adequately supported by the results?

Partly

Are sufficient details of methods and analysis provided to allow replication by others?

Yes

Reviewer Expertise:

Reproductive physiology, Male contraception, infertility diagnosis, cancer diagnosis, endocrinology

I confirm that I have read this submission and believe that I have an appropriate level of expertise to confirm that it is of an acceptable scientific standard.

## References

[1] : Evaluation of selected semen parameters and biomarkers of male infertility – preliminary study. *F1000Research* .2022;11: 10.12688/f1000research.121622.1 10.12688/f1000research.121622.1 38434001 PMC10905134
